# The voltage-gated proton channel Hv1 contributes to neuronal injury and motor deficits in a mouse model of spinal cord injury

**DOI:** 10.1186/s13041-020-00682-6

**Published:** 2020-10-20

**Authors:** Madhuvika Murugan, Jiaying Zheng, Gongxiong Wu, Rochelle Mogilevsky, Xin Zheng, Peiwen Hu, Junfang Wu, Long-Jun Wu

**Affiliations:** 1 Department of Neurology, Mayo Clinic, Rochester, MN 55905 USA; 2 Department of Cell Biology and Neuroscience, Rutgers University, Piscataway, NJ 08854 USA; 3 One Harvard Street Institute of Health, Brookline, MA 02446 USA; 4 Department of Anesthesiology, University of Maryland, Baltimore, MD 21201 USA; 5 Department of Neuroscience, Mayo Clinic, Jacksonville, FL 32224 USA; 6 Department of Immunology, Mayo Clinic, Rochester, MN 55905 USA

**Keywords:** Microglia, Spinal cord injury, Hv1, Voltage-gated proton channel, ROS, Neuroinflammation, Interleukin-1β

## Abstract

Traumatic injury to the spinal cord initiates a series of pathological cellular processes that exacerbate tissue damage at and beyond the original site of injury. This secondary damage includes oxidative stress and inflammatory cascades that can lead to further neuronal loss and motor deficits. Microglial activation is an essential component of these secondary signaling cascades. The voltage-gated proton channel, Hv1, functionally expressed in microglia has been implicated in microglia polarization and oxidative stress in ischemic stroke. Here, we investigate whether Hv1 mediates microglial/macrophage activation and aggravates secondary damage following spinal cord injury (SCI). Following contusion SCI, wild-type (WT) mice showed significant tissue damage, white matter damage and impaired motor recovery. However, mice lacking Hv1 (*Hv1*^*−/−*^) showed significant white matter sparing and improved motor recovery. The improved motor recovery in *Hv1*^*−/−*^ mice was associated with decreased interleukin-1β, reactive oxygen/ nitrogen species production and reduced neuronal loss. Further, deficiency of Hv1 directly influenced microglia activation as noted by decrease in microglia numbers, soma size and reduced outward rectifier K^+^ current density in *Hv1*^*−/−*^ mice compared to WT mice at 7 d following SCI. Our results therefore implicate that Hv1 may be a promising potential therapeutic target to alleviate secondary damage following SCI caused by microglia/macrophage activation.

## Highlights

- Microglial activation after spinal cord injury is regulated by voltage-gated proton channel Hv1.
- Mice lacking Hv1(*Hv1*^*−/−*^) display reduced cytokine production and oxidative stress responses.
- *Hv1*^*−/−*^ mice exhibit improved pathology and motor recovery outcomes following spinal cord injury.

## Introduction

Injury to the spinal cord initiates a complex cascade of oxidative stress and inflammatory responses at and beyond the original site of insult and aggravates tissue damage [1]. These secondary cascades that occur from minutes to weeks after primary injury, can lead to further demyelination, glial scar formation, and additional neuronal loss [2]. Hence, therapeutic intervention during the window of secondary injury is key to influence the tissue and behavioral recovery following spinal cord injury (SCI).

Following SCI, microglia activation occurs within minutes and is subsequently accompanied by infiltration of blood-borne monocytes into lesion area at 3–7 d after injury through chemokine signaling [3, 4]. These monocytes differentiate into phagocytic macrophages [5], which along with microglia contributes to the innate immune response including production of inflammatory cytokines, debris clearing and tissue repair [2, 3, 6]. The beneficial versus harmful role of microglia/macrophage in SCI remains debated [2]. For instance, microglia/macrophages are the source of pro-inflammatory cytokines such as interleukin-1β (IL-1β), interleukin-6, tumor necrosis factor-α, nitric oxide, and reactive oxygen species (ROS), which are considered to be neurotoxic and growth inhibitory [7–9]. In contrast, injury-activated microglia and macrophages were shown to be essential for clearance of damaged/degenerating tissue, wound compaction and motor recovery after SCI [10, 11]. Thus far microglia and macrophage activation has been well-studied in SCI, however, the underlying signaling mechanisms remain unclear.

The voltage-gated proton channel Hv1, encoded by gene *Hvcn1*, is known to be expressed exclusively on microglia in the healthy central nervous system (CNS), and some immune cells such as neutrophils and macrophages in the periphery [12–14]. As an efficient proton channel regulating cytoplasmic pH, Hv1 couples to NOX2-dependent ROS generation during pathologies. We previously showed that, mice lacking Hv1 (*Hv1*^*−/−*^) had reduced brain damage and alleviated motor deficits compared to wild-type (WT) mice in a mouse model of middle cerebral artery occlusion (MCAO) and photothrombotic (PT) ischemia [12, 15, 16]. These rescued phenotypes in *Hv1*^*−/−*^ mice in ischemic stroke were accompanied by reduced ROS production and shifted microglial polarization from M1 to M2 state [15]. Interestingly, Hv1 was also implicated in cuprizone-induced microglial oxidative damage and subsequent demyelination [17]. In line with this, a recent study showed that *Hv1*^*−/−*^ mice had significantly reduced expression of chondroitin sulfate proteoglycans (CSPGs), decreased oligodendrocyte apoptosis and cavity formation after SCI compared to WT mice [18]. However, whether Hv1 is essential for microglial activation and implicated in motor recovery after SCI remains unknown.

Our study implicates Hv1 in microglia activation and secondary damage following moderate spinal cord contusive injury. Most importantly, Hv1 deficiency (*Hv1*^*−/−*^) rescued SCI-induced increase in oxidative stress resulting in enhanced neuronal survival, white matter sparing, and improved locomotive recovery. Together, the current study suggests that Hv1 is a novel target candidate to reduce secondary injury following SCI.

## Results

### Mice with Hv1 deficiency exhibit reduced motor deficits and tissue damage following SCI

**Fig. 1:**
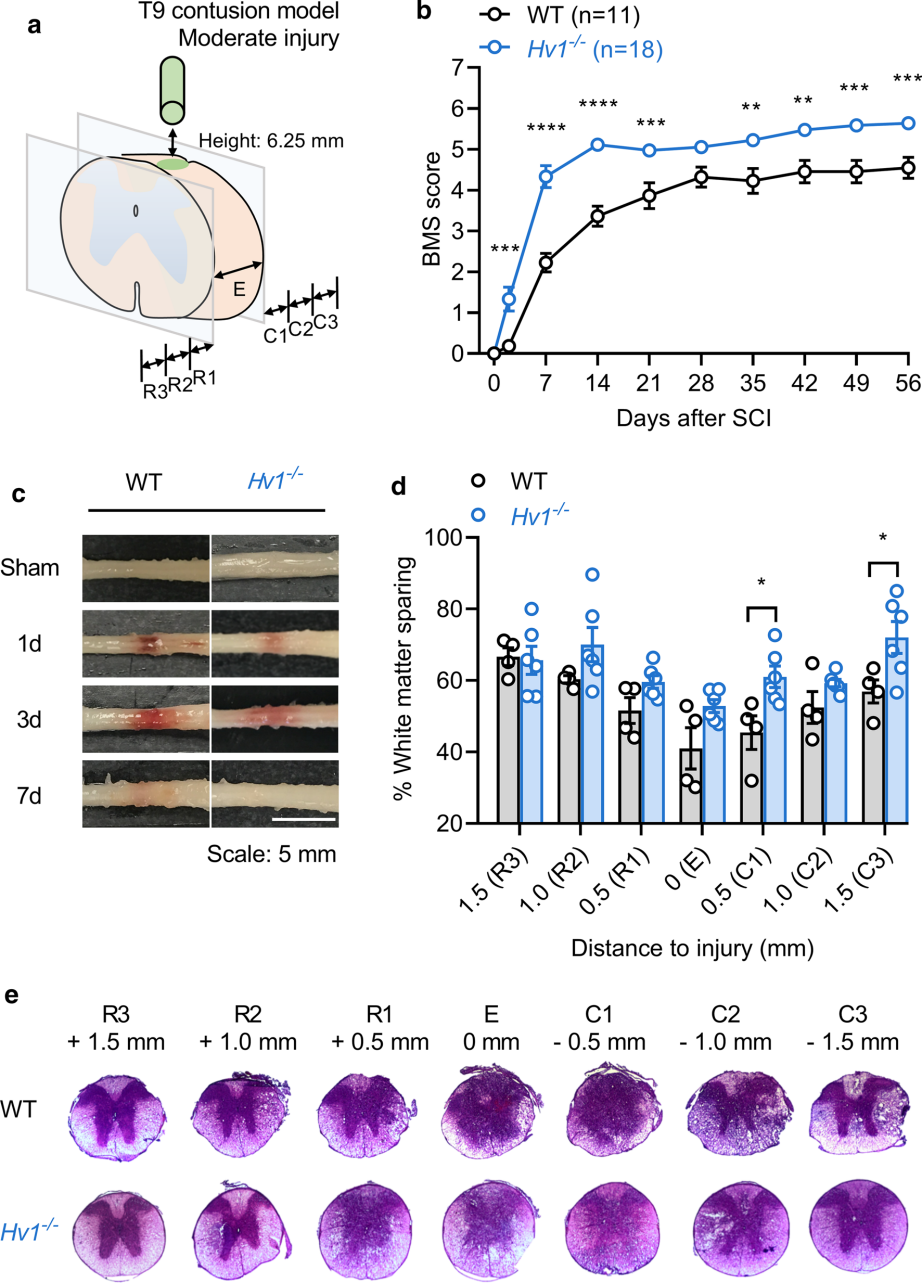
Genetic deletion of Hv1 results in better locomotor recovery after SCI. **a** Schematic showing injury site and regions investigated for white matter sparing in this study. **b** Total BMS scores in WT and *Hv1*^*−/−*^ mice measured at different time points following SCI and sham controls (WT, *n* = 11; *Hv1*^*−/−*^, *n* = 18). *Hv1*^*−/−*^ mice show significantly improved locomotor recovering from day 2 (*P < 0.05, **P < 0.01, ***P < 0.001, two-way ANOVA with repeated measures). **c** Representative images of perfused spinal cord from WT and *Hv1*^*−/−*^ mice from sham, 1 d, 3 d and 7 d after SCI (Scale: 5 mm). **d** Bar graph denotes the percentage of white matter area out of total spinal cord section area, represented as mean ± SEM. **e** Representative images of H&E stained spinal cord sections at different locations from the epicenter of injury obtained from WT and *Hv1*^*−/−*^ mice at 7 d after SCI. (WT, *n* = 4; *Hv1*^*−/*−^, *n* = 6; *P < 0.05, **P < 0.01, ***P < 0.001, two-way ANOVA

To investigate the role of Hv1 in SCI, we employed the mouse spinal cord contusion injury model. WT and *Hv1*^*−/−*^ mice were subjected to moderate contusion injury at the ninth thoracic vertebra (T9). The location of injury and the sagittal planes tested for further analysis are shown in Fig. 1a. All mice subjected to SCI lost the motility of hind limbs in the beginning (Fig. 1b). We noted that WT mice do not have any motor function recovery at 2 d post injury, whereas *Hv1*^*−/−*^ mice exhibit slight or extensive ankle movement during BMS locomotion testing. At 7 d post injury, significant mobility function recovery was observed between WT mice (only ankle movement) and *Hv1*^*−/−*^ mice (occasional or frequent plantar stepping, no coordination, or some coordination), and the significant difference persists till the end of the 8 week observation period (Fig. 1b and Additional file 1: Figure S1A). To separate the results from male and female mice, we also note that there are no significant sex differences when we compared *Hv1*^*−/−*^ mice to WT mice after SCI (Additional file 1: Figure S1B and C). The BMS locomotion testing results implicate that Hv1 may play a critical role in the secondary damage especially during the early stage of SCI, and the alteration of secondary damage from early time points may impact locomotor recovery.

Since significant recovery differences were noted in the early phase following SCI, we then assessed whether the improvement in motor recovery of *Hv1*^*−/−*^ mice was associated with reduced secondary tissue damage after SCI. Perfused spinal cord tissues showed *Hv1*^*−/−*^ mice have reduced hemorrhage around lesion areas following SCI (Fig. 1c). Moreover, the lesion area stained with H&E revealed enhanced myelin sparing in *Hv1*^*−/−*^ mice compared with WT mice at 7 d after SCI (Fig. 1d, e). Prevention of myelin loss in *Hv1*^*−/−*^ mice is evident around the lesion epicenter and at distances up to 1500 µm rostral and caudal (R3 & C3) to the epicenter (Fig. 1d, e). Although the most significant damage to white matter is noted at the epicenter (Fig. 1e), the tissue recovery in the rostro-caudal locations away from the injury (R1-R3, C1-C3) tend to vary. Previous studies using non-invasive imaging tools such as diffusion tensor imaging show that axon demyelination and necrosis occurs rostrally to the lesion in the ascending tracts and caudally to the lesion in the descending tracts and may be led by Wallerian degeneration [19]. This might explain the variation in location of white matter sparing noted in our model. Since hemorrhaging and white matter loss are key indicators of the process of secondary injury [20], the result strongly suggested that deficiency of Hv1 can alleviate the secondary damage at the early stage following SCI. Therefore, our further study was focused on 1 d, 3 d and 7 d after SCI to determine how Hv1 mediates secondary injury in the early stage of injury.

### Hv1 mediates microglia activation following SCI

**Fig. 2:**
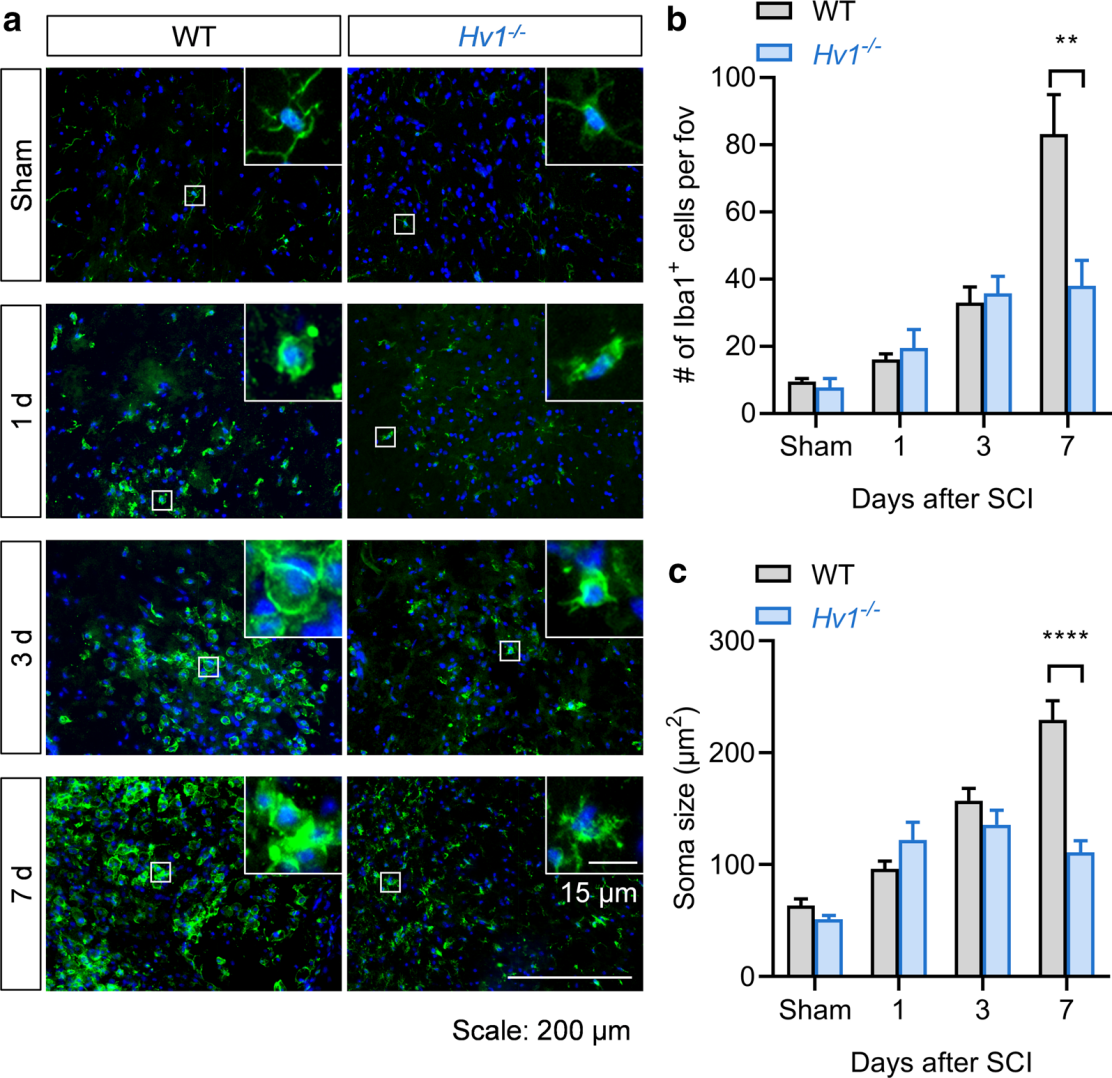
Microglial activation is reduced in *Hv1*^*−/−*^ mice after SCI. **a** Representative images showing time course of microglial activation (Iba1) in *Hv1*^*−/−*^ and WT mice after SCI (Scale: 200 µm). Insets show higher magnification images of single Iba1^+^ cell from the lower magnification image (Scale: 15 µm). **b, c** Bar graph denotes number and soma size of Iba1^+^ cells per field of view (fov) in ventral horn in sham, 1 d, 3 d and 7 d in both WT and *Hv1*^*−/−*^ mice. Data is represented as mean ± SEM. (*P < 0.05, **P < 0.01, ***P < 0.001, Student’s *t*-test, *n* = 3–5 per group)

During CNS traumatic injury, microglia are the first responders followed by astrocyte activation and recruitment of the peripheral immune cells, such as neutrophils and macrophages [3, 21, 22]. To address the question how Hv1 mediates microglial responses post SCI, we employed immunofluorescence staining of ionized calcium binding adaptor molecule 1 (Iba1) to assess microglia cell number and soma size change post SCI in WT and *Hv1*^*−/−*^ mice. Both WT and *Hv1*^*−/−*^ mice have increased number of Iba1^+^ cells and increased soma size after SCI (Fig. 2a–c). However, the Iba1^+^ cell number and soma in *Hv1*^*−/−*^ mice plateaus at 3–7 d, while increases continuously in WT mice (Fig. 2b, c), suggesting that depletion of Hv1 may alleviate microglia/macrophage activation after SCI.

**Fig. 3:**
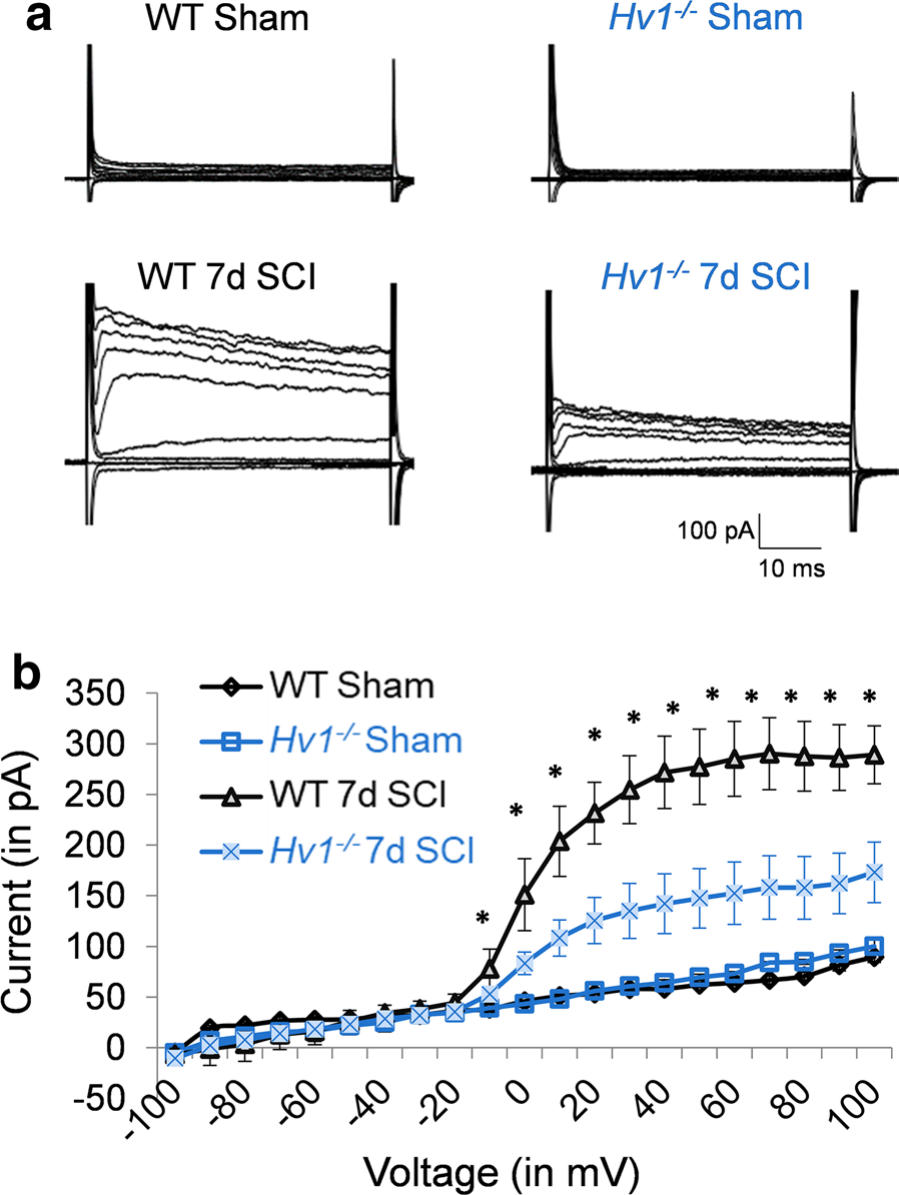
Microglia membrane currents is reduced in *Hv1*^*−/−*^ mice after SCI. **a** Membrane currents of microglia in WT and *Hv1*^*−/−*^ mice in sham and 7 d after SCI mice. **b** Summarized plot of current (pA) versus voltage (mV) in microglia in WT and *Hv1*^*−/−*^ mice after SCI and in their corresponding sham mice. Data is represented as mean ± SEM. (*P < 0.05 compared against respective sham control, Student’s *t*-test, *n* = 5 per group)

In addition to the cell density and morphological analysis, we employed whole cell patch clamp recordings to test the functional activation of microglia by measuring their membrane currents from live spinal cord slices in sham and 7 d after SCI in WT and *Hv1*^*−/−*^ microglia. Microglia are labeled with CX3CR1-GFP. We found that outward potassium current exhibits linear current–voltage in microglia at both WT and *Hv1*^*−/−*^ sham groups. Whereas, an outward rectifying current was noted at 7 d after SCI in both WT and *Hv1*^*−/−*^ microglia, but the current in WT microglia is significantly higher than that of in *Hv1*^*−/−*^ microglia at 7 d after SCI (Fig. 3a, b), suggesting WT microglia is more activated than *Hv1*^*−/−*^ microglia after SCI. Together, our data shows that microglia are both morphologically and electrophysiologically less activated in *Hv1*^*−/−*^ mice compared with WT mice after SCI.

### Hv1 mediates pro-inflammatory response at the lesion area

**Fig. 4:**
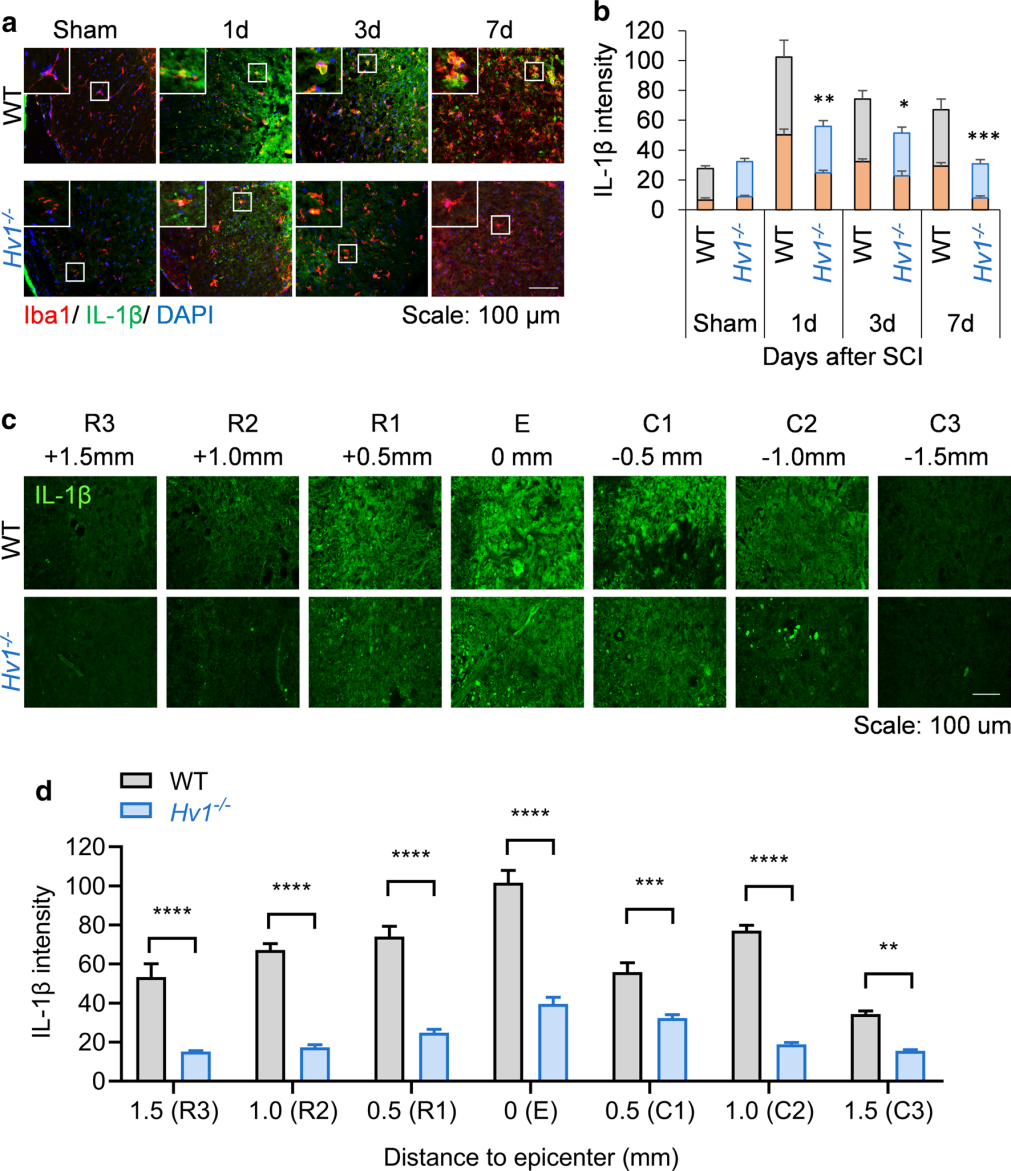
IL-1β expression is reduced in *Hv1*^*−/−*^ mice after SCI. **a** Representative images showing colocalization of IL-1β (green) with Iba1 (red) in the white matter of WT and *Hv1*^*−/−*^ mice in sham, 1 d, 3 d and 7 d after SCI (Scale: 100 um). **b** Bar graph shows intensity of IL-1β staining in the sham and SCI groups in WT (gray) and *Hv1*^*−/−*^ (blue) mice, and percentage of IL-1β staining co-localizing with Iba1 (orange). **c** Representative images of IL-1β staining in ventral horn at various sagittal planes (R3-C3) along the spinal cord in WT and *Hv1*^*−/−*^ at 3 d after SCI (Scale: 100 um). **d** Bar graph denotes IL-1β staining intensity of R3-C3 in WT and *Hv1*^*−/−*^ at 3 d after SCI and is represented as mean ± SEM. (*n* = 3, *P < 0.05, **P < 0.01, ***P < 0.001, Student’s *t*-test)

We further investigate whether Hv1 depletion alters microglial functions during secondary damage after SCI. Interleukin-1β (IL-1β) was reported to be highly expressed following SCI [23] and is an indicator of secondary damage [24]. IL-1β is well-known pro-inflammatory cytokine aggregating inflammatory response and expressed highly in activated microglia [23]. To investigate IL-1β expression pattern in WT and *Hv1*^*−/−*^ mice, we performed immunofluorescence staining and found IL-1β production is significantly reduced in *Hv1*^*−/−*^ mice (Fig. 4a, b). Furthermore, the co-localization staining of IL-1β and Iba1^+^ cells manifested that microglial expression of IL-1β peaks at 1 d after SCI in both WT and *Hv1*^*−/−*^ mice, and loss of Hv1 reduces the IL-1β production (Fig. 4b). It is important to note that reduced IL-1β in *Hv1*^*−/−*^ mice may be a direct effect of reduced Iba1^+^ cells. On the other hand, IL-1β staining in ventral horn at various sagittal planes (R3–C3) along the spinal cord shows that loss of Hv1 not just reduces IL-1β production, but also makes the pro-inflammatory response more limited to the lesion epicenter (Fig. 4c, d). However interestingly, when we employed cytokine array to investigate how Hv1 alters cytokine/chemokine production on a broad scale at 3 d after SCI, IL-1β does not stand out. It could be explained that at 3 d after SCI, IL-1β production is reduced, also it is diluted in 5 mm-tissue homogenates (Additional file 2: Figure S2). We noted a significant increase in expression of CXCL13, C5/C5a, CXCL10, M-CSF, CCL2, CCL12, CXCL12, TIMP-1, TNFα, TREM-1 after SCI in WT, which was prevented in *Hv1*^*−/−*^ mice, with the exception of IL-1rα which was further upregulated in *Hv1*^*−/−*^ compared to WT mice at 3 d after SCI (Additional file 2: Figure S2).

### Loss of Hv1 reduces oxidative stress damage and attenuates neuronal loss

**Fig. 5:**
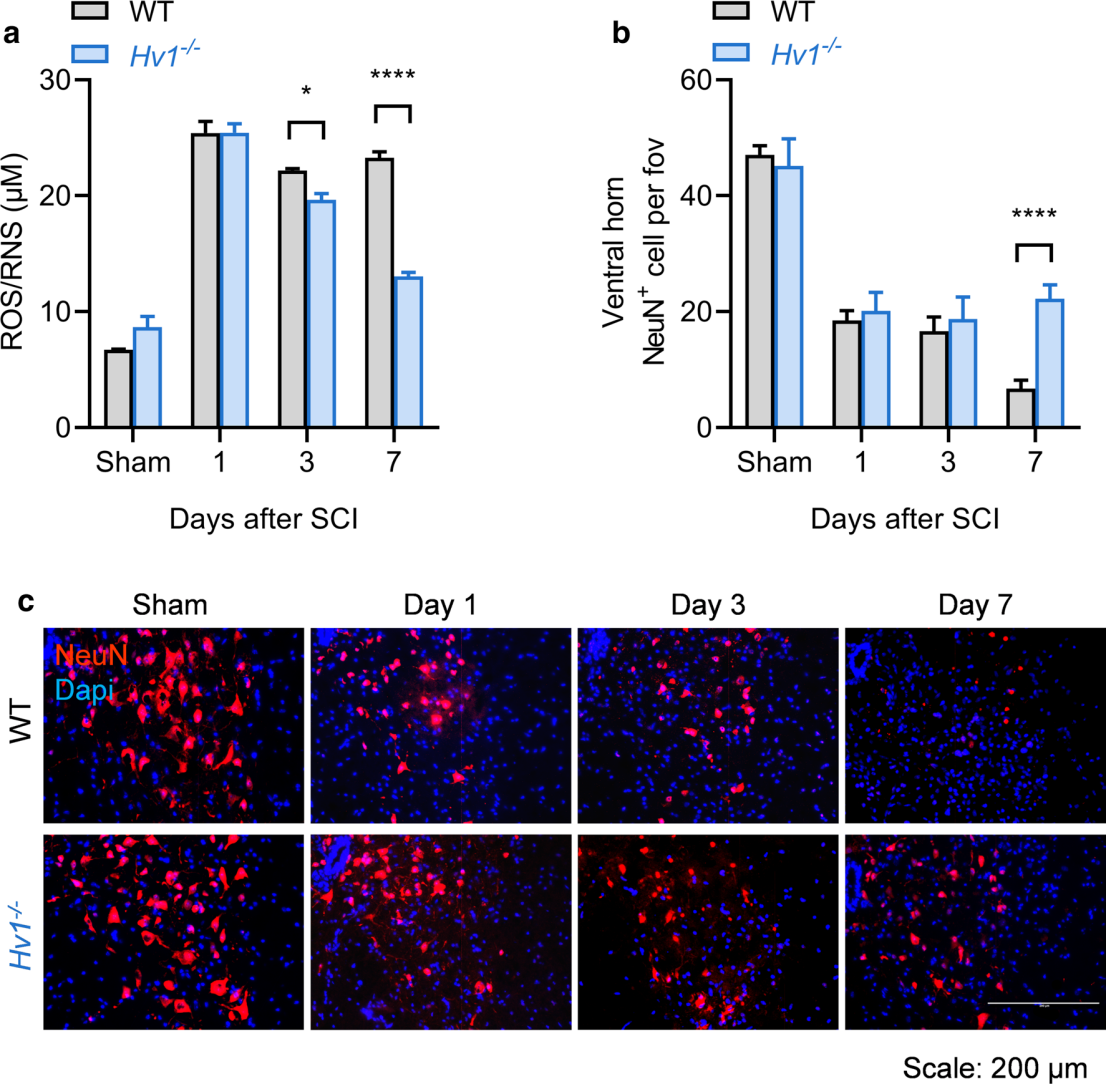
*Hv1*^*−/−*^ mice show reduced oxidative stress induced damage to neurons. **a** Bar graph shows ROS content in spinal tissue homogenates measured by the OxiSelect™ ROS/RNS assay kit in the sham and SCI groups in WT (gray) and *Hv1*^*−/−*^ (blue) mice. **b** Analysis of number of NeuN^+^ cells per field of view (fov) in the ventral horn of the spinal cord in sham, 1 d, 3 d and 7 d after injury. **c** Representative images show time course of neuronal loss (NeuN) in *Hv1*^*−/−*^ and WT mice after SCI (Scale: 200 µm). Data is represented as mean ± SEM. (*P < 0.05, **P < 0.01, ***P < 0.001, Student’s *t*-test, comparison between WT and *Hv1*^*−/−*^ for each time point, *n* = 3 per group/ time point)

Previous work reported that microglial Hv1 is coupled with NOX2 for inflammatory responses in ischemia stroke, and depletion of Hv1 can efficiently inhibit NOX-mediated ROS damage [12]. To investigate whether Hv1 also mediates ROS production after SCI, we employed ROS/RNS assay to detect the amount of ROS/RNS in the region containing injured epicenter. ROS/RNS level is low in both sham groups and no significant difference found between WT and *Hv1*^*−/−*^ mice (Fig. 5a). After SCI, the concentration of ROS/RNS peaks in both WT and *Hv1*^*−/−*^ mice at 1 d, and the amount of ROS/RNS production in *Hv1*^*−/−*^ mice significantly decreased compared to WT at 3 d and 7 d after SCI, respectively (Fig. 5a). We next investigated whether increased and extended release of ROS contributes to secondary damage after SCI. As a readout, we employed immunofluorescence staining to quantify the number of survived NeuN^+^ neurons in the lesion area. Both WT and *Hv1*^*−/−*^ mice experience severe neuronal loss after SCI, however while the number of motor neurons continuously decreases in WT mice, the neuronal loss was attenuated in *Hv1*^*−/−*^ mice (Fig. 5b, c). These findings suggest that Hv1 deficiency reduces oxidative stress during the secondary damage and results in attenuated neuronal loss.

## Discussion

**Fig. 6:**
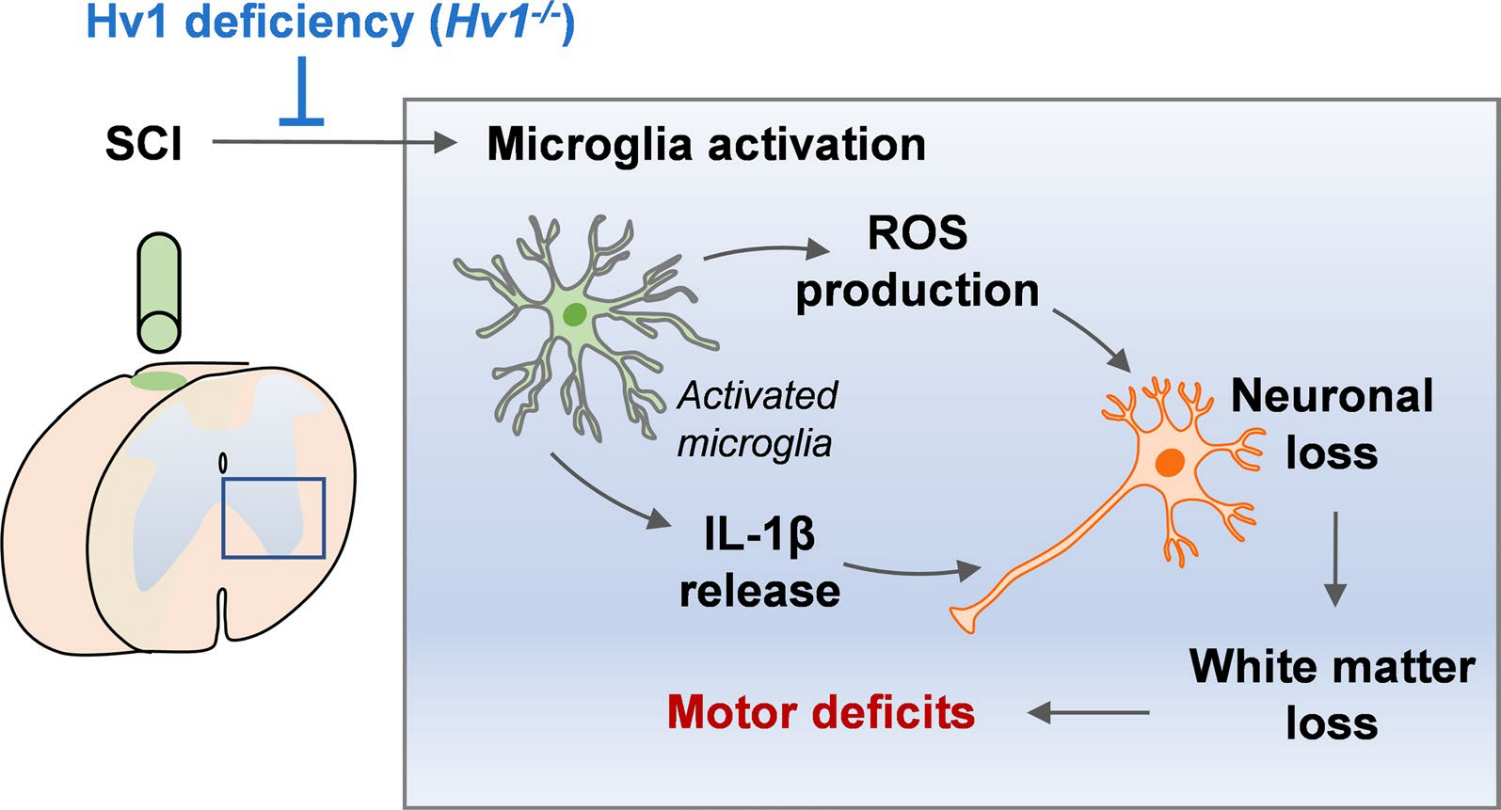
Schematic figure of microglia Hv1-mediated neuronal injury and motor deficits following SCI. The illustration shows that SCI leads to Hv1-dependent microglia activation which causes an increase in IL-1β and ROS production. Consequently, there is an increase in neuronal loss, white matter loss and motor dysfunction. Deficiency of Hv1 prevents microglia activation and thereby ameliorates IL-1β / ROS production, neuronal injury and resultant motor deficits seen following SCI

In the present study, we show that Hv1 deficiency is sufficient to rescue motor deficits caused by SCI. The Hv1-mediated changes in motor deficits are linked to microglia activation, IL-1β release, ROS production and neuronal loss (Fig. 6). In addition to resident microglia, a number of circulating immune cell types respond to SCI [3, 21, 22]. It is noteworthy that the infiltrating cell types such as macrophages and neutrophils also express Hv1, similar to the resident microglial population [25–27] and may contribute to SCI pathology seen in our study. Thus, future study is needed to determine the respective contributions of neutrophils and macrophages to Hv1-mediated ROS damage following SCI. To that end, conditional Hv1 mice are necessary to address these important questions on cell type specific Hv1 function in vivo.

Loss of white matter in and around the injury site is believed to be the main underlying cause for the subsequent motor deficits. A study investigating the anatomical correlates of locomotor recovery after spinal lesions showed that even a small proportion of spared white matter can mediate large effects on functional recovery after SCI [28]. Moreover, lesion localization is crucial, with white matter sparing in the ventrolateral affecting open-field locomotion [28]. In line with this, we noted significant white matter sparing in the ventral horn of the spinal cord and corresponding motor deficits following SCI, which is rescued in *Hv1*^*−/−*^ mice. It has been noted that oligodendrocyte death inducing axonal demyelination are major contributors to white matter damage and represents the major pathogenesis after SCI [29]. Interestingly, we previously showed that Hv1 deficiency promotes oligodendrocyte survival in an experimental model of multiple sclerosis [17]. A recent study also showed that deletion of Hv1 attenuated apoptosis of oligodendrocytes, and ameliorated myelin loss after SCI, all of which correlated with significant tissue repair [18]. These results are consistent with our studies that show improved white matter sparing and reduced neuronal loss in *Hv1*^*−/−*^ mice after SCI.

Expression of Hv1 is associated with microglial activation toward M1 polarization [12, 15, 18]. In both an MCAO‑induced and a PT-induced model of ischemic stroke, large Hv1-mediated currents were detected in microglia, but not in neurons or astrocytes [12, 15]. Microglia membrane currents are representative of ion channel expression pattern, which strongly depends on the cells' microenvironment and has been recognized as a sensitive marker of the cells' functional state [30, 31]. Here, we noted that microglia outward rectifier K^+^ current was significantly larger at 7 d after SCI in WT mice compared to *Hv1*^*−/−*^ mice. These membrane currents are typical of voltage-gated Kv1.3 channel activation and are distinct from the H^+^ currents that are mediated by Hv1 channel activation [16]. In our previous work, we showed that H + currents are completely abolished in mice [16]. However, whether there is a direct link between Hv1 and Kv1.3 channel activation *Hv1*^*−/−*^ need to be examined. We believe that increased Hv1 activation in microglia after SCI led to increased ROS/cytokine production indirectly resulting in Kv1.13 channel activation. In support of this, Hv1 upregulation in the aged brain shifted the dynamic equilibrium of microglial activation toward M1 polarization and exaggerated post-operative neuroinflammatory responses after peripheral surgical intervention [32]. Since the outward rectifying K^+^ currents are required for microglial pro-inflammatory activation in vivo [33], this explains the inverse link between IL-1β/cytokine production and Hv1 expression noted in our study.

Microglia activation in terms of morphological changes manifests only around 7 d after SCI, however, it is possible that microglia/macrophages are already generating ROS via Hv1 activation at earlier time points (3 d). In fact, it has been noted in many instances that stimulation of microglia/macrophages does not lead to overt activation, but rather primes the cellular NOX for enhanced ROS production [34–36]. Our data confirms that *Hv1*^*−/−*^ mice generate reduced ROS at 3 d after SCI well before microglia morphological changes are noted, suggesting that ROS generation via Hv1-NOX axis may precede microglia activation and possibly explains the functional improvement observed at early time points.

The differential roles of resident microglia and infiltrated macrophages have gained increased attention in many pathological conditions, particularly in SCI [8, 37, 38]. Newly developed strategies to specifically eliminate microglia, such as the use of PLX5622, a CSF1R inhibitor, demonstrated that the absence of microglia disrupts the organization of the astrocytic scar, reduces the number of neurons and oligodendrocytes at the site of injury, and impairs functional recovery after SCI [39]. Using targeted fate mapping, another recent study showed that microglia within the spinal lesion site respond heterogeneously, forming multiple activation states [40]. Further, the activated microglia can prevent the infiltration of peripheral macrophages and thereby prevent further white matter damage [40]. These studies suggest that microglia are neuroprotective in the context of SCI. However, our results suggest that Hv1 activation after SCI in microglia/macrophages is harmful to neurons via the release of pro-inflammatory cytokines and ROS. The dual function of microglia has been reported in a variety of neurological disorders such as stroke, epilepsy, and neurodegeneration, depending on the context and timing of the disease progression [41–43]. The differential regulation of Hv1-mediated ROS production by blood-derived macrophages compared to resident microglia [44] may contribute to the neurotoxic effects of Hv1 activation noted in this study. Another important, yet-unrecognized role for Hv1 in SCI, is its role in regulation of phagosomes in innate immune cells such as microglia, macrophages and neutrophils [13]. It has been previously shown that microglia efficiently phagocytose damaged tissue and remain viable, whereas, macrophages of peripheral origin are less efficient at processing debris, and their death, in situ, may contribute to the secondary damage after CNS injury [11]. Additional mechanism may also include that Hv1 as a proton releasing channel which could activate the acid-sensing ion channel to induce neurotoxicity [14, 16, 45], or Hv1-mediated microglia-astrocyte interaction [46]. Taken together, the differential mechanisms mediated by Hv1 in microglia versus macrophages in microglia polarization, ROS production, maintenance of phagosomes, and proton signaling might affect the course and outcome of SCI and needs to be elucidated.

In summary, our study demonstrates the role of Hv1 in SCI-induced motor deficits. Further, it implicates Hv1 in microglia/macrophage activation, white matter damage, IL-1β/ cytokine production, ROS generation and neurona loss after SCI. Insights into the Hv1-mediated mechanisms may aid therapeutic designs for ameliorating microglia/macrophage activation and improve motor recovery after SCI.

## Materials and methods

### Animals

All experiments were conducted using adult male C57BL/6 J mice or *Hv1*^*−/−*^ mice (20–30 g). For whole-cell patch clamp recordings, *Hv1*^*−/−*^*:CX3CR1*^*GFP/*+^ mice were generated by breeding *CX3CR1*^*GFP/GFP*^ mice with *Hv1*^*−/−*^ mice, the resulting *Hv1*^*+/−*^*:CX3CR1*^*GFP/*+^ offspring were in turn crossbred with *Hv1*^*−/−*^ mice, and only mice with *Hv1*^*−/−*^*:CX3CR1*^*GFP/*+^ genotype were used for the experiments. Female mice were only used to investigate the role of sex difference in locomotor recovery. All mice were housed on a 12:12 h light/dark cycle with food and water available ad libitum. The experiments were performed in accordance with institutional guidelines, as approved by the animal care and use committee at Rutgers University and Mayo Clinic. The animals were monitored on a daily basis after SCI. Any unintended changes in appearance (disheveled hair, weight loss, and dehydration), behavior (decreased grooming, eating and drinking) and activity (decreased exploring and nesting) were noted and euthanized if no improvement was observed.

### SCI contusion model and post-surgery animal care

The spinal cord contusion injury was performed as described previously [47]. Briefly, mice were anesthetized with ketamine (60 mg/kg, i.p.) and xylazine (10 mg/kg, i.p.) and a laminectomy over the dorsal portion of T9. The spinal column was stabilized via the lateral processes using clamps at T8 and T13 vertebrae. The exposed dorsal surface of the spinal cord was subjected to a 3 g weightdrop with tip diameter of 0.5 mm flat surface, from a height of 6.25 mm using an impactor resulting in a moderate contusion injury [47]. After the injury, a small piece of subcutaneous fat was placed on the dorsal surface before the skin was closed, in order to avoid tissue adhesion between dura and peripheral tissues. For sham control, laminectomy was performed without injuring spinal cord. After SCI, mice were subcutaneously injected with 0.1 ml 0.125% (50 μl per 25 g body weight of mice) bupivacaine near the surgical area 5 min after surgery for post-operative analgesia. 0.5 ml Saline (SAL-090; Rocky Mountain Biologicals) and 0.05 ml 20 mg/ml cefazolin (054,846; Henry Schein) were subcutaneously injected daily for 3 days. Mice were kept on absorbent bedding and their bladders were manually expressed twice each day until a reflex bladder was established (14–28 d after SCI).

### Locomotion recovery assessment

Mice were tested for locomotor recovery of the hindlimbs in an open-field chamber on day 2 after injury and weekly thereafter for up to 8 weeks using the Basso mouse scale (BMS) for locomotion [48]. Successful contusion surgery resulted in complete motor function loss of the hind limbs 2 h after surgery impact (BMS score 0, no ankle movement), whereas successful sham surgery models had no effect on hind limb mobility (BMS score 9, frequent or consistent plantar stepping, mostly coordinated, paws parallel at initial contact and lift off, normal trunk stability, and tail up).

### Whole-cell patch clamp recording

Live spinal cord slices were prepared at 7 d after SCI in WT (*CX3CR1*^*GFP/*+^ mice) and *Hv1*^*−/−*^ (*Hv1*^*−/−*^* CX3CR1*^*GFP/*+^) mice or in sham controls [31, 49]. Briefly, mice were anesthetized in isoflurane and perfused with ice-cold artificial cerebrospinal fluid (ACSF). The spinal cord was swiftly removed and the slices (300 µm) were made using a vibratome in ice-cold oxygenated (95% O_2_ and 5% CO_2_) ACSF with the following composition (in mM): NaCl, 124; NaHCO_3_, 25; KCl, 2.5; KH_2_PO_4_, 1; CaCl_2_, 2; MgSO_4_, 2; glucose, 10 and sucrose added to make 300–320 mOsmol. The slices were then transferred to a recovery chamber for 30 min with oxygenated ACSF with the same composition as above at room temperature before electrophysiological studies. Whole cell patch-clamp recordings were made on microglia with *CX3CR1*^*GFP/*+^ transgencially labeled (~ 50 μm deep). Recording electrodes (4 –5 MΩ) contained a K-based internal solution composed of (in mM): 120 K-gluconate, 5 NaCl, 1 MgCl_2_, 0.5 EGTA, 10 Na_2_Phosphocreatine, and 10 HEPES (pH 7.2; 280–300 mOsmol). The membrane potential was held at −20 mV. Data were amplified and filtered at 2 kHz by a patch-clamp amplifier (Multiclamp 700B), digitalized (DIGIDATA 1440A), stored, and analyzed by pCLAMP (Molecular Devices, Union City, CA). Data were discarded when the input resistance changed > 20% during recording. The voltage ramp test was performed from −100 to 100 mV in 500 ms. For electrophysiology, a minimum of five cells from at least three different mice from the same litter were randomly selected for recording per condition.

### Tissue preparation

Mice were deeply anaesthetized by isoflurane and perfused transcardially with 40 ml of cold phosphate-buffered saline (PBS) followed by 40 ml of cold 4% paraformaldehyde (PFA) in PBS [50]. The spinal cord was removed and post-fixed with the same 4% PFA for 6 h at 4 °C, then transferred to 30% sucrose in PBS for 48 h. Tissues were embeded in optimal cutting temperature compound (OCT compound) prior to frozen sectioning. Cryosection was performed to collect complete 2–4 mm region containing the injuried epicenter. Sample Sects. (15 mm in thickness) were prepared on gelatin-coated glass slide with a cryostat (Leica) for further staining.

### White matter sparing analysis

Hematoxylin–eosin (H&E) staining was used to examine white matter sparing after SCI as described previously [51]. Thermo Scientific™ Shandon™ Rapid-Chrome H & E Frozen Section Staining Kit was applied on spinal cord frozen sections according to manufacturer’s instructions (9990001, Thermo Scientific™). Frozen spinal cord sections mounted on glass slides were allowed to acclimate to room temperature for at least 30 min, and then washed with tris buffered saline (TBS) for 10 min. According to staining protocol, the slides were placed in Rapid-Fixx solution for 5–7 s. Then slides were dipped into distilled water for 10 times. After that, slides were incubated in hematoxylin for 1 min, dipped into distilled water for another 10 times, and dipped 3 times in bluing reagent. Enriched positive charges allowed hematoxylin to bind DNAs, and turned nucleus into blue. Slices were then dehydrated in 95% alcohol by 5–7 dips, followed by 15 s in Eosin-Y solution, which stained cytoplasm with red or pink. Finally, slices were dipped 5–7 times into 95% alcohol, 100% alcohol, 100% alcohol again, xylene, and xylene again, in turn, for the purpose of dehydration and clearing. The slides were imaged with a bright-field microscope. Grey matter had a denser population of cells (nucleus) compared to the white matter, thus H&E staining enhanced the contrast between grey and white matter. The perimeter of the white matter was traced and the enclosing area was measured using ImageJ software (National Institutes of Health, Bethesda, MD).

### Immunofluorescence staining

Frozen spinal cord sections mounted on glass slides were allowed to acclimate to room temperature for at least 30 min, and then washed with TBS to remove OCT compound. The slices were blocked with 5% goat serum in 0.4% Triton X-100 in TBS for 60 min, and then incubated overnight at 4 °C with the following primary antibodies: rabbit-anti-Iba1 antibody (1:500; 019–19,741; Wako, Japan); rabbit anti-NeuN (1:1000; ab104225; Abcam, USA); mouse anti-IL-1β (1:300; 12,242; Cell Signaling, USA). After primary antibody incubation, slides were re-warmed to room temperature for 30 min and washed with TBS three times for 15 min each wash. Then slices were incubated for 90 min at room temperature with secondary antibody in the dark (1:500, Alexa Fluor 594 goat anti-rabbit, Alexa Fluor 488 goat anti-mouse, Life Technologies). The sections were mounted with DAPI Fluoromount-G (SouthernBiotech) for further imaging.

### Quantitative image analysis

Fluorescent images were obtained with a fluorescence microscope (EVOS FL Cell Imaging System, Life Technologies). Cell counting, soma size analysis and fluorescent signal intensity were quantified using ImageJ software (National Institutes of Health, Bethesda, MD). For the quantification of microglia and macrophages (Iba1^+^) and neurons (NeuN^+^), the total number of immunolabeled cells stained with nuclear marker DAPI was counted at 20 × magnification images. For the quantification of IL-1β, imaging parameters were kept constant to avoid technical artefacts in the analysis of fluorescent signal intensity.

### Reactive oxygen species assay

ROS content in spinal tissue homogenates were measured using the OxiSelect™ ROS/RNS assay kit (Cell Biolabs, STA- 347) according to the manufacturer’s instructions. In brief, mice were perfused transcardially with 40 ml cold PBS. Approximately 16 mg fresh spinal cord samples containing the SCI epicenter were extracted and homogenized with tissue extraction reagent I (10 ml PER 1 g of tissue; Invitrogen FNN0071) with protease and phosphatase inhibitors, and then supernatant was collected after centrifugation. Lysate was added to the wells along with, dichlorodihydrofluorefscin (DCF), a ROS specific fluorescent probe in the presence of a catalyst. The resultant fluorescent intensity was proportional to the amount of free radicals in the sample. The assay was performed in a 96-well plate format using a standard fluorescence plate reader. The free radical content in samples was determined by comparison with the predetermined DCF standard curve.

### Cytokine array

Proteome Profiler Mouse Cytokine Array Kit (R&D systems, ARY006) was applied to assess the inflammatory response according to manufacturer’s instructions [52]. In brief, mice were anesthetized by isoflurane and perfused transcardially with ice-cold PBS. Protein samples were prepared in the same way as for ROS assay and 300 μg of proteins collected from the spinal cord was used for each sample and were run as duplicates.

### Statistical analysis

All data are presented as the mean ± SEM and were analyzed for significant differences between groups by two-way ANOVA with post hoc Tukey test for multiple comparisons. In all cases, *P* < 0.05 was considered to be significant.

## Supplementary information

**Additional file 1: Figure S1.** Deficiency of Hv1 shows better motor recovery in both males and females. A. BMS sub-scores in WT and *Hv1*^*−/−*^ mice (males and females combined) at different time points following SCI and sham controls (WT, n = 11; *Hv1*^*−/−*^, n = 18). B. Total BMS scores and C. BMS sub-scores in WT and *Hv1*^*−/−*^ mice measured in males and females at different time points following SCI (WT: males, n = 8; females, n = 3; *Hv1*^*−/−*^: males, n = 13; females, n = 5). (*P < 0.05, **P < 0.01, ***P < 0.001, two-way ANOVA with repeated measures).

**Additional file 2: Figure S2.** *Hv1*^*−/−*^ mice have distinct cytokines/chemokine expression pattern after SCI. A. Schematic representation of the cytokine array. The array contains 40 different antibodies to mouse cytokines/chemokines, three positive controls (PC) and one negative control (NC), all in duplicates (upper right). The representative immunoblots of cytokines/chemokines in the spinal cord lysates (WT sham control, upper right; WT 3 d after SCI, bottom left; *Hv1*^*−/−*^ 3 d after SCI, bottom right) are shown. B. Bar graph denotes optical density representing the expression level of cytokines/chemokines. Data is represented as mean ± SEM. (n = 3, * signifies comparison between Sham and WT 3 d after SCI. # signifies comparison between WT and *Hv1*^−/−^ 3 d after SCI, *,^#^P < 0.05, **,^##^P < 0.01, ***, ^###^P < 0.001, Student’s t-test).

## Data Availability

The datasets are available from the corresponding author on reasonable request.
