# Supplementary figures and images for: The voltage-gated proton channel Hv1 contributes to neuronal injury and motor deficits in a mouse model of spinal cord injury
Source: Mol Brain. 2020 Oct 20;13:143. doi: 10.1186/s13041-020-00682-6 (PMC7574559; doi:10.1186/s13041-020-00682-6)

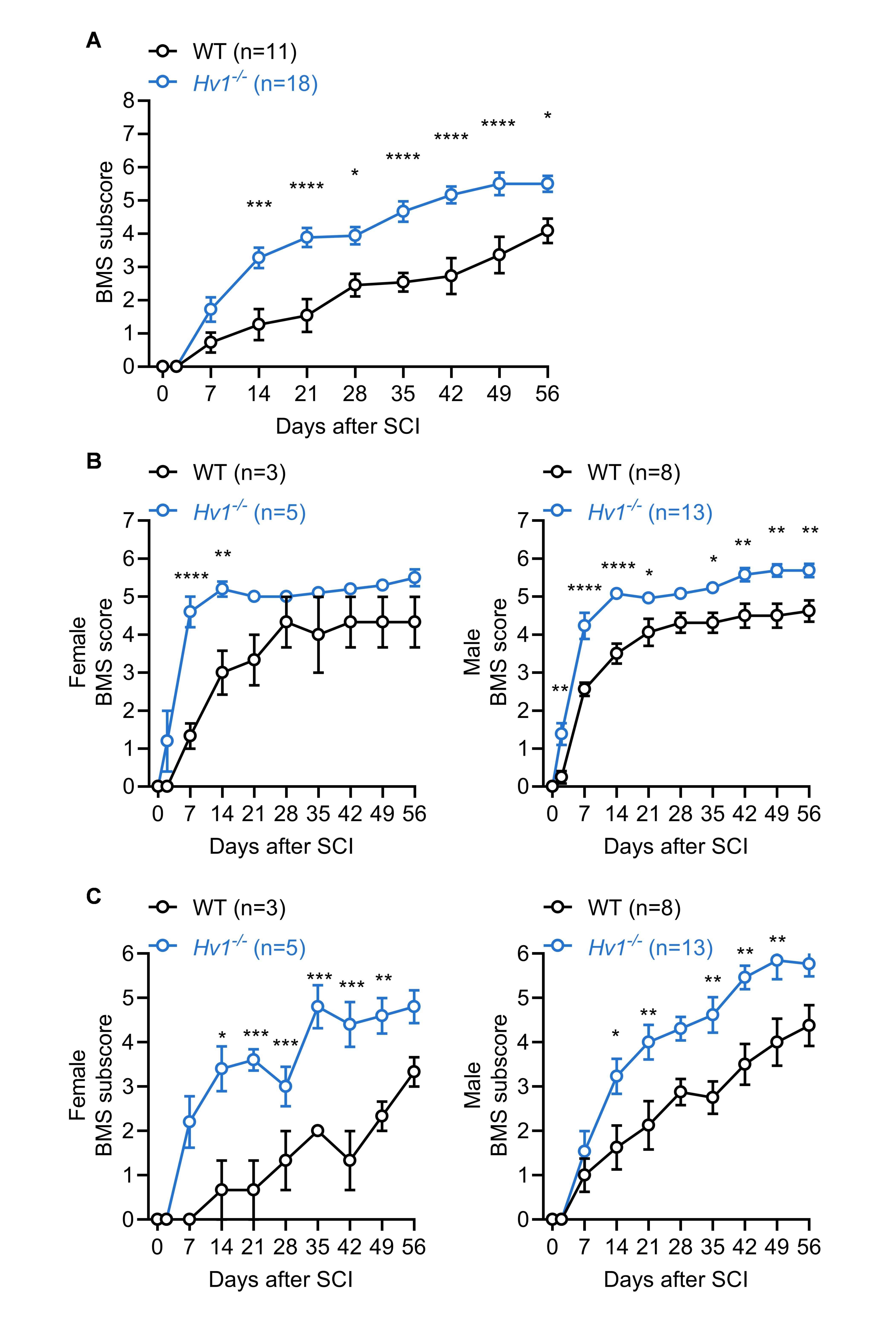

Supplement: Supplementary file 1 — Additional file 1: Figure S1. Deficiency of Hv1 shows better motor recovery in both males and females. A. BMS sub-scores in WT and Hv1−/− mice (males and females combined) at different time points following SCI and sham controls (WT, n = 11; Hv1−/−, n = 18). B. Total BMS scores and C. BMS sub-scores in WT and Hv1−/− mice measured in males and females at different time points following SCI (WT: males, n = 8; females, n = 3; Hv1−/−: males, n = 13; females, n = 5). (*P < 0.05, **P < 0.01, ***P < 0.001, two-way ANOVA with repeated measures). [file 13041_2020_682_MOESM1_ESM.tif]

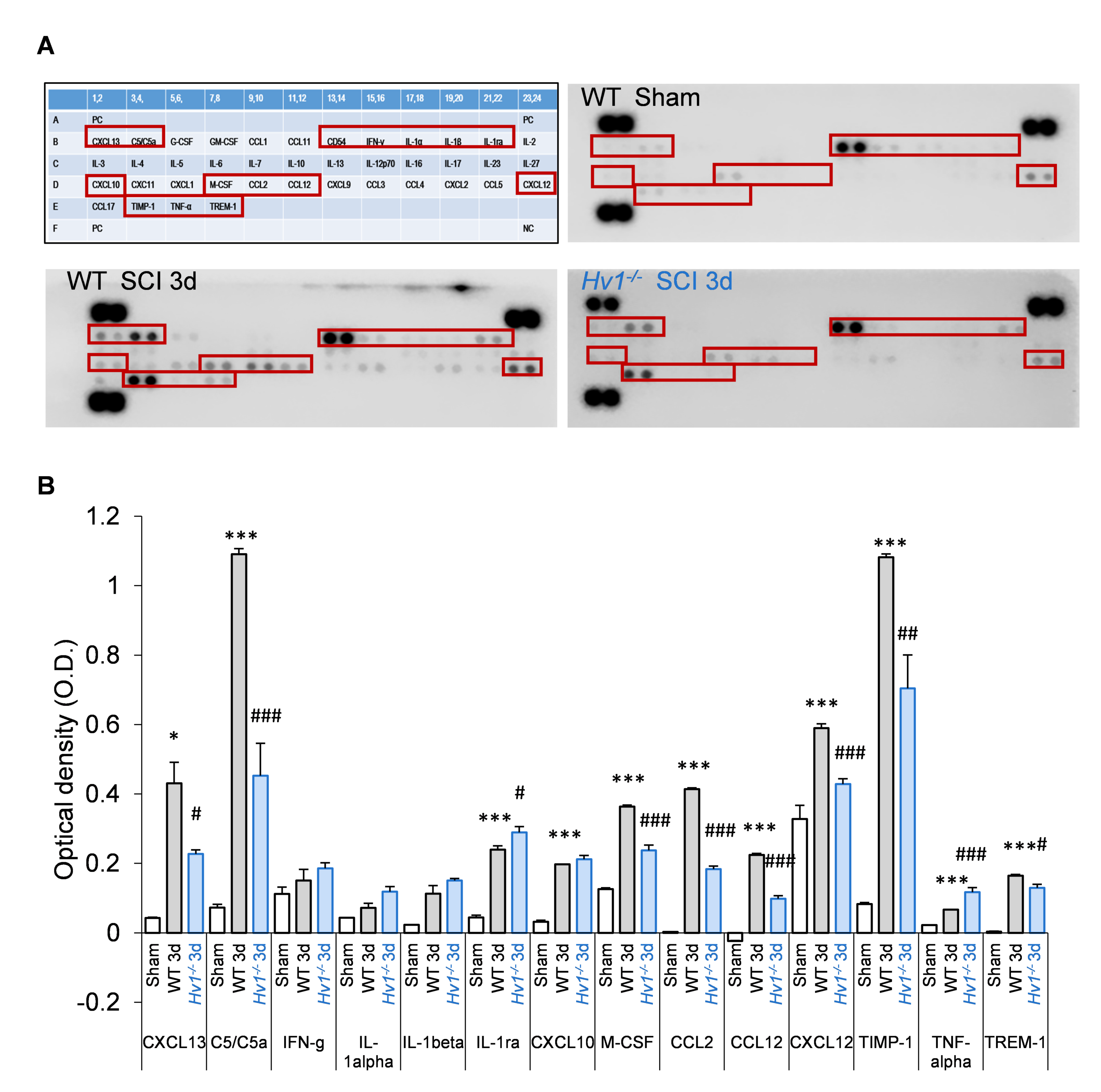

Supplement: Supplementary file 2 — Additional file 2: Figure S2. Hv1−/− mice have distinct cytokines/chemokine expression pattern after SCI. A. Schematic representation of the cytokine array. The array contains 40 different antibodies to mouse cytokines/chemokines, three positive controls (PC) and one negative control (NC), all in duplicates (upper right). The representative immunoblots of cytokines/chemokines in the spinal cord lysates (WT sham control, upper right; WT 3 d after SCI, bottom left; Hv1−/− 3 d after SCI, bottom right) are shown. B. Bar graph denotes optical density representing the expression level of cytokines/chemokines. Data is represented as mean ± SEM. (n = 3, * signifies comparison between Sham and WT 3 d after SCI. # signifies comparison between WT and Hv1−/− 3 d after SCI, *,#P < 0.05, **,##P < 0.01, ***, ###P < 0.001, Student’s t-test). [file 13041_2020_682_MOESM2_ESM.tif]
